# Transcriptome Changes Affecting Hedgehog and Cytokine Signalling in the Umbilical Cord: Implications for Disease Risk

**DOI:** 10.1371/journal.pone.0039744

**Published:** 2012-07-10

**Authors:** Walter Stünkel, Hong Pan, Siew Boom Chew, Emilia Tng, Jun Hao Tan, Li Chen, Roy Joseph, Clara Y. Cheong, Mei-Lyn Ong, Yung Seng Lee, Yap-Seng Chong, Seang Mei Saw, Michael J. Meaney, Kenneth Kwek, Allan M. Sheppard, Peter D. Gluckman, GUSTO Study Group, Joanna D. Holbrook

**Affiliations:** 1 Singapore Institute for Clinical Sciences, Agency for Science Technology and Research, Singapore, Singapore; 2 Yong Loo Lin School of Medicine, National University of Singapore, National University Health System, Singapore, Singapore; 3 Saw Swee Hock School of Public Health, National University of Singapore, National University Health System, Singapore, Singapore; 4 Department of Maternal Fetal Medicine, KK Women’s and Children’s Hospital, Singapore, Singapore; 5 Liggins Institute, University of Auckland, Auckland, New Zealand; VU University Medical Center, The Netherlands

## Abstract

**Background:**

Babies born at lower gestational ages or smaller birthweights have a greater risk of poorer health in later life. Both the causes of these sub-optimal birth outcomes and the mechanism by which the effects are transmitted over decades are the subject of extensive study. We investigated whether a transcriptomic signature of either birthweight or gestational age could be detected in umbilical cord RNA.

**Methods:**

The gene expression patterns of 32 umbilical cords from Singaporean babies of Chinese ethnicity across a range of birthweights (1698–4151 g) and gestational ages (35–41 weeks) were determined. We confirmed the differential expression pattern by gestational age for 12 genes in a series of 127 umbilical cords of Chinese, Malay and Indian ethnicity.

**Results:**

We found that the transcriptome is substantially influenced by gestational age; but less so by birthweight. We show that some of the expression changes dependent on gestational age are enriched in signal transduction pathways, such as Hedgehog and in genes with roles in cytokine signalling and angiogenesis. We show that some of the gene expression changes we report are reflected in the epigenome.

**Conclusions:**

We studied the umbilical cord which is peripheral to disease susceptible tissues. The results suggest that soma-wide transcriptome changes, preserved at the epigenetic level, may be a mechanism whereby birth outcomes are linked to the risk of adult metabolic and arthritic disease and suggest that greater attention be given to the association between premature birth and later disease risk.

## Introduction

Birth outcomes defined by gestational age and birth weight have far reaching consequences across the life-course. For instance young adults born prematurely or at very low birthweights have significantly lower bone density than do their larger and term-born peers [1]. The prenatal environment has been linked to the risk of disease in later life [2], [3], [4], [5], [6], [7], [8]. Children born either prematurely or small for gestational age have reduced insulin sensitivity and are at higher risk for type 2 diabetes mellitus [7], [8]. Little is known of the molecular mechanisms by which these longer-term consequences for the offspring are transmitted, although epigenetic mechanisms appear to be implicated [9]. Umbilical cord tissue is a readily available tissue and offers a source of RNA and DNA for an assessment of the genomic and epigenomic state of the neonate. Molecular biomarkers that reveal early life experience and predict later disease risk would be valuable as a means to identify high-risk patients, and could be a starting point to designing therapeutic interventions [9], [10], [11]. While several groups have found transcriptomic [12], [13] and epigenetic marks [14], [15], [16], [17], [18], [19], [20], [21] in the umbilical cord associated with extreme intrauterine experience or birth outcomes, of particular interest to us and relevant to global health concerns are the molecular mechanisms of inter-individual variability operating within the normal range [22], as long-term effects of the prenatal state can be readily demonstrated within children of normal birthweight and gestation [9], [23], [24].

GUSTO (Growing Up in Singapore Towards healthy Outcomes) is a Singapore-based birth cohort study [25]. From this deeply phenotyped cohort we profiled the transcriptome of 32 umbilical cords collected at birth from ethnic Chinese babies in Singapore hospitals. Transcriptomic change significantly related to gestational age were discovered and then validated by q-PCR analysis across an expanded set of 127 multiethnic samples. Pathway analysis revealed enrichment of differential transcription by gestational age in hedgehog signalling genes: GLI2, GLI3, and SMO, and others downstream of the pathway with a role in skeletal development, defects of which are associated with early-onset osteoarthritis. In addition, inflammatory mediators such as CXCL14 and Il1RL1 were differentially expressed, as well as HSD11B1, a gene with known implications in development [26]. Furthermore, a subset of the differentially expressed genes was found to also differ in DNA methylation levels in a manner associated with gestational age. We suggest that lower birth weight and particularly earlier (within the near term range) gestational ages, leaves an epigenetic echo that affects the risk for diseases such as type 2 diabetes mellitus and osteoarthritis in later life.

## Results

**Figure 1:**
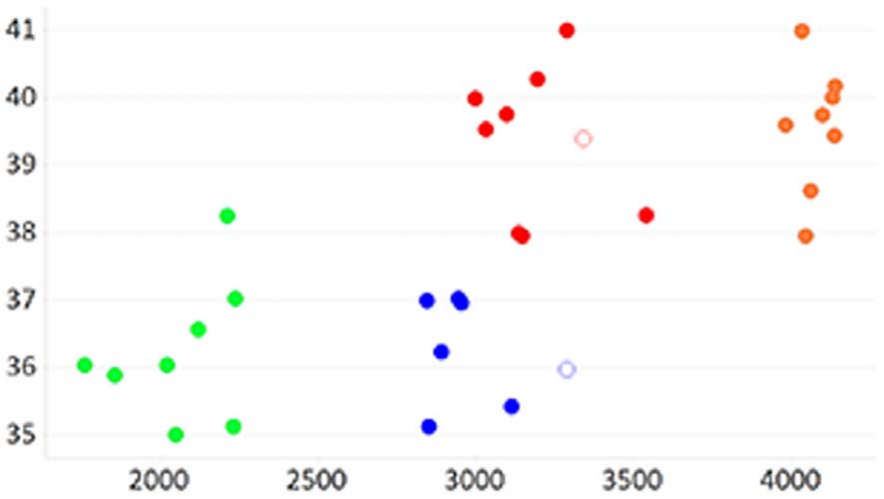
RNA Expression Microarray Study Design. Gestational age in weeks (y-axis) and birth-weight in grams (x-axis) of the samples analysed by expression microarrays are symmetrical to allow somewhat independent comparisons for birth-weight and gestational age. Samples are classified into high birth weight group (>3700 g) in orange; low birthweight group (<2500 g) in green; normal birthweight and gestational age less than or equal to 37 weeks in blue; or normal birthweight and gestational age more than 37 weeks in red. Two samples that failed QC are shown as non-filled circles.

To ascertain the independent roles of both gestational age and birth weight, which are positively correlated, we selected normal birth weight samples to match the gestational ages of the extreme birth weight samples [Figure 1 and Table 1], and subjected them to expression microarray analysis. After probe quality control and inter-sample quantile normalisation of the 32 RNA samples interrogated on the array, the data for two samples failed quality control (MAD scores <−5) and were removed from subsequent analyses. The removed samples were from the ≤37 week_NBW and from the >37 w_NBW groups and are highlighted in Figure 1. Three series of technical replicates were included in the experiment, they clustered together in 10 cases out of 12; when the full dataset was subjected to unsupervised hierarchical clustering demonstrating that the intra-sample variation is lower than the inter-sample variation [Figure S1]. Therefore the data were declared of acceptable quality and the replicates were combined.

**Table 1:** Demographic and Characteristics of the Participants.

		RNA expression array (n = 32)		PCR (n = 120)		DNA methylation array (n = 20)	
		N (%)	Mean (SD)	N (%)	Mean (SD)	N (%)	Mean (SD)
Gender	Female	16 (50%)		51 (43%)		11 (55%)	
	Male	16 (50%)		69 (58%)		9 (45%)	
GA (weeks)	≤37 weeks	14 (44%)	36 (1)	9 (8%)	36 (1)	10 (50%)	36 (1)
	>37 weeks	18 (56%)	39 (1)	111 (93%)	39 (1)	10 (50%)	40 (1)
BW (g)	LBW (<2500)	8 (25%)	2056 (1799)	24 (20%)	2280 (125)	5 (25%)	2069 (202)
	NBW (2500–3700)	16 (50%)	3099 (191)	67 (56%)	3141 (320)	10 (50%)	3046 (153)
	HBW (>3700)	8 (25%)	4076 (62)	29 (24%)	4073 (319)	5 (25%)	4086 (51)
Ethnic Group	Chinese	32 (100%)		58 (48%)		20 (100%)	
	Malay			37 (31%)			
	Indian			25 (21%)			
Parity			0.6 (1)		0.9 (1)		0.6 (1)
Maternal age		34 (4)	34 (4)		31 (5)		35 (3)

**Figure 2:**
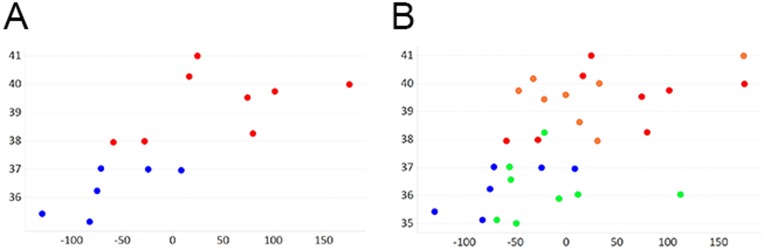
The largest source of variation in the transcriptomics data is associated with gestational age across samples. Prinicipal component analysis using the RNA expression microarray data across just the normal birthweight samples (A) or across all samples (B), returned principal component 1 (x-axis) which has a significant correlation with gestational age of the samples. Samples are classified into high birth weight group (>3700 g) in orange; low birthweight group (<2500 g) in green; normal birthweight and gestational age less than or equal to 37 weeks in blue; or normal birthweight and gestational age more than 37 weeks in red.

To examine the pattern of relatedness between individual transcriptomes and to identify potential underlying associations with birth outcomes, principal component analysis was performed on the dataset. We found that within the normal birth weight samples, PC1 separated the samples by gestational age (*p*<0.05, R = 0.76) [Figure 2A]. When extreme birth weight samples were included in the analysis, there was still a separation by gestational age, but the correlation was not as strong (*p*<0.05, R  = 0.55) [Figure 2B]. However there was no significant relationship (p<0.05) with birth weight, gender or maternal age for any of the first five components returned from the principal component analysis. Therefore we concluded that the majority of the signal in the dataset is driven by gestational age and not by birth weight, suggesting that for infants born within the normal range of birth weights and gestational ages, gestational age is a stronger driver of umbilical cord transcriptomes than is birth weight.

Next, we utilized linear regression to identify which probe expression levels were most highly correlated with gestational age, and then determine if any associated with birth weight. As predicted from the above, we found more probes for which expression correlated with gestational age, (530 had a *p*-value <0.001) [Table S1] than with birthweight (8 had a *p*-value <0.001) [Table S2]. Some of the probes most strongly correlated with gestational age mapped to hedgehog ligand *GLI2* [Figure S2A] and hedgehog receptor smoothened, *SMO* [Figure S2B]. Figure S3 shows the correlation of expression of a probe mapping to transforming growth factor receptor beta 1 (*TGFBR1*) with birthweight.

ANOVA tests were performed to identify gene expression associated with gestational age, by comparing the normal birth weight samples from the two different gestational age groups delimited by 37 weeks (i.e. ≤37 w_NBW vs. >37 w_NBW), 64 probes passed an FDR correction for multiplicity of q<0.05 [Table S3]. Other probes with nominally significant p-values (<0.05) (not passing FDR) and outlier fold changes included those mapping to desmocollin 1 (*DSC1*), a cadherin protein involved in epithelial cell adhesion in desmosomes [27], which had the highest fold change [Figure S2C]. We also performed an ANOVA on all samples grouped by gestational age regardless of birthweight, and *DSC1* still had the one of the highest fold changes [Figure S2D].

**Figure 3:**
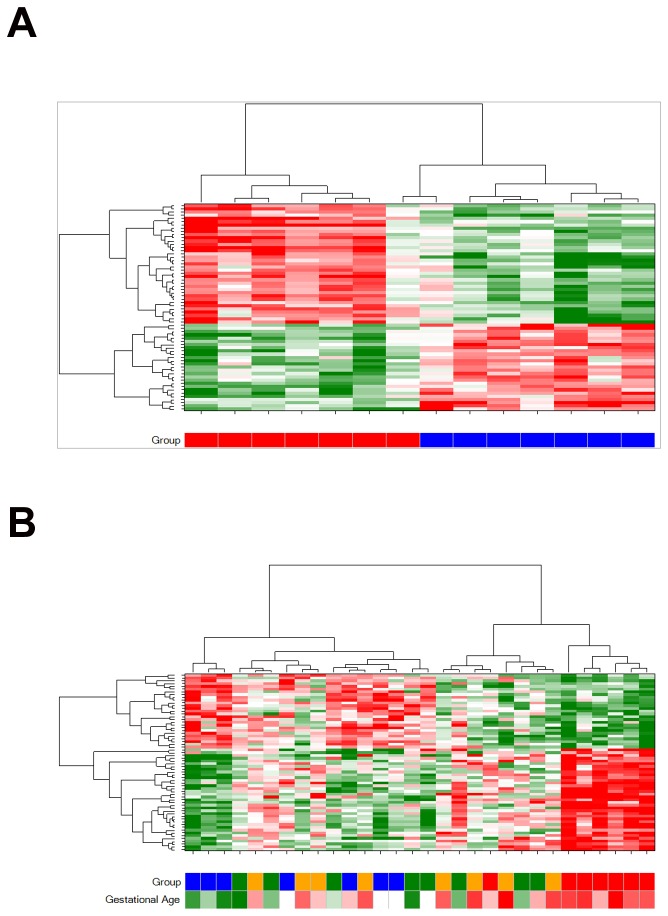
Expression signature for gestational age organises samples into gestational age groups. Hierarchical clustering of samples (columns) by the expression levels of the 64 probes (rows) significantly associated with gestational age (adjusted p-value<0.05), organises normal birth weight samples perfectly by gestational age group (A) and organises all samples into two clusters with significantly different gestational ages (B). Z-score normalised logged expression levels are denoted in the heat map (green for low, red for high, white for intermediate). X-axis colour bars denote sample classification: high birth weight group (>3700 g) in orange; low birthweight group (<2500 g) in green; normal birthweight and gestational age less than or equal to 37 weeks in blue; or normal birthweight and gestational age more than 37 weeks in red. Gestational age is also represented as a continuous variable in the x-axis colour bar in (B) green for low, red for high, white for intermediate.

The 64 probes which passed FDR in the gestational age comparison were capable of organising the samples by gestational age when used in hierarchical clustering. Clustering on just the normal birth weight samples gave the cleanest result [Figure 3A]. However, when the extreme birth weight samples were included, the bifurcating pattern still significantly separated the samples in respect to their gestational age (average gestational age of left branch  = 36.96 weeks, average gestational age of right branch  = 39.12 *t*-test *p*-value  = 0.0003) [Figure 3B].

We also performed ANOVAs to identify genes for which transcript levels were differential for birth weight, by comparing the low birth weight (LBW) group with the ≤37 w_NBW group; and the high birth weight (HBW) group with the >37 w_NBW group. No probes passed an FDR correction for multiplicity (adjusted p<0.05), in either comparison. Eight probes corresponding to five known genes co-varied with BW (p<0.05) and had p<0.05, fc>1.5 in both ANOVA tests [Table S4 and Figure S3].

**Figure 4:**
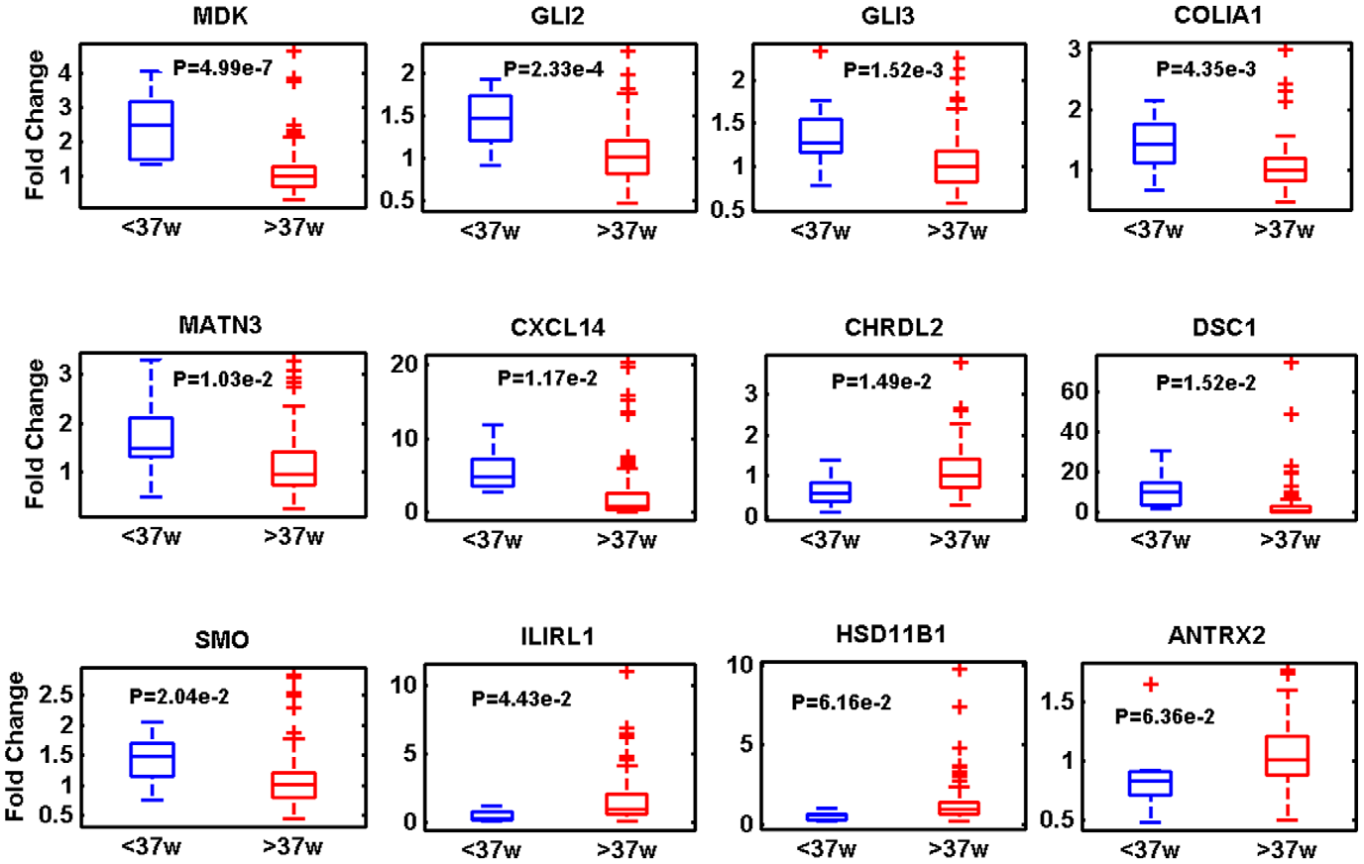
Twelve transcripts have differential expression levels in gestational age groups across the 120 sample replication set. Fold change with regard to the median sample of the more than 37 weeks gestation group, is shown on the y-axis. Gene names are shown above each panel. P-values from the 2 group tests are shown within each panel. Data is represented as a box plot where the 2–3 quartile range is within the box, the median is denoted by a horizontal line within the box, the min and max are denoted by horizontal lines outside of the box and single outliers are represented by crosses.

We selected some of the strongest expression changes along with some of the more interesting genes from a disease-related perspective and assayed their expression levels in 127 additional samples. Twenty-two genes in all were tested with a range of significance in the array data (Table S5). Generally there was a concordance between the magnitude of the fold change in the ANOVA and repetition in the expansion set (average absolute fold change replicating group (shown in figure 4 and table S5)  = 2.52, average absolute fold change non-replicating group (shown in table S5)  = 1.47, *t*-test p = 0.026). The replicating group comprises genes whose mRNA levels had a significant relationship with gestational age in the qPCR expanded study. The non-replicating group are those genes whose mRNA levels did not achieve significance against gestational age in the expanded qPCR study. Of this set, 12 genes showed p<0.1 in a t-test between the less than 37 weeks gestational age (<37 w) and more than 37 weeks gestational age (>37 w) groups, they are illustrated in Figure 4.

Pathway enrichment analysis was performed on the 45 genes mapping to the 64 probes which had FDR <0.05 for gestational age. The most enriched gene ontology (GO) process was skeletal system development (Fisher’s exact test p-value = 8.4e^−5^), with 4/129 genes related to this processes showing significant changes: *MATN3*, *MSX1*, *PITX1* and *BMP8B*.

**Figure 5:**
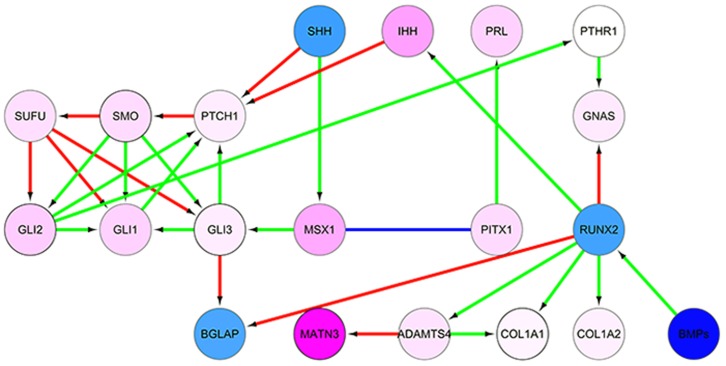
Subnetwork enriched for differential expression by gestational age. Nodes represent genes and are coloured by the fold change of their transcripts by gestational age (blue for positive association with gestational age, pink for negative association). No probe for *PTHR1* was included on the array. Arrows represent literature-verified interactions and the colours denote the type of interaction (green for activation, red for inhibition and blue for co-expression).

Pathway enrichment analysis was also performed on 445 genes mapping to the 530 probes co-varying with gestational age (p<0.001, by univariate regression). The most enriched GeneGO map was “Hedgehog and PTH signalling pathways in bone and cartilage development” (Fisher’s exact test p-value = 3.3e^−4^) with 5/24 genes for the pathway showing significant changes: *SMO*, *GNAS*, *GLI2*, *COL1A1* and *COL1A2*. We also noted that the expression of *GLI3*, another central component of the hedgehog pathway, was correlated with gestational age. Components of hedgehog signalling and bone development pathways have been shown to interact with each other, and we were able to generate a subnetwork of genes enriched for differential expression for gestational age [Figure 5]. For most of the genes in figure 5, transcript levels were higher in the ≤37 week gestation group.

Schroeder et al (2011) [28] showed that the DNA methylation profile of umbilical cords reflects gestational age. Therefore, we surveyed the DNA methylome of a representative subset of twenty, of the samples originally examined by gene expression microarrays. Of the twelve genes with validated differential expression by gestational age (Figure 4), eight genes (67%) had concordant differential CpG methylation levels by gestational age (Pearson covariate <0.05) [Table 2]. Of note were the three canonical members of the Hedgehog signalling pathway *GLI2, GLI3* and *SMO*, which showed methylation at multiple CpGs varying with gestational age. A specific CpG dinucleotide in the 5′UTR of *GLI3* (cg17530977) was one of the 41 CpGs whose methylation levels reached experiment-wide significance for gestational age in Schroeder et al (2011) [28]. While this CpG was not significant in our study, other similarly located CpGs close to the 5′UTR, transcription start site and within the gene body suggest coherent results with Schroeder et al’s findings. Intriguingly we found a significant correlation across samples for a subset of CpGs in table 2, both by comparing the values within each sample with no regard to phenotype and by comparing the ratios between differential gestational age pairs (Text S1). Similarly, we also considered DNA methylation changes in the eight genes showing the most significant differential expression for birthweight, and found 4 (33%) with significant methylation differences by birthweight [table S6].

**Table 2:** Genes whose differential transcription for gestational age was replicated across the qPCR groups, also containing CpGs whose methylation levels correlated with gestational age.

Gene Name	CpG ID	CpG location	UCSC_RefGene_Group	Correlation PValue	min β-value	max β-value	Min-max range	Average β for lowest GA samples	Average β for highest GA samples	Range between highest and lowest GA
CHRDL2	cg13616912	Island	TSS1500	0.0184	4%	9%	6%	8%	5%	3%
COL1A2	cg18511007		TSS200	0.0123	3%	13%	10%	8%	5%	3%
CXCL14	cg23510026	S_Shore	TSS1500	0.0163	86%	95%	8%	90%	93%	3%
GLI2	cg18835090		Body	0.0039	80%	88%	8%	80%	85%	5%
GLI2	cg23854087		Body	0.0292	78%	87%	8%	80%	83%	3%
GLI2	cg13713922	S_Shore	Body	0.0367	7%	16%	9%	14%	9%	4%
GLI2	cg07855121		Body	0.0203	92%	97%	5%	93%	96%	3%
GLI2	cg15471519		Body	0.0476	73%	80%	7%	75%	76%	1%
GLI3	cg00842259	N_Shore	Body	0.0494	78%	87%	9%	83%	87%	4%
GLI3	cg21536074		Body	0.0101	81%	90%	9%	89%	82%	7%
GLI3	cg01013023		Body	0.0268	84%	96%	12%	87%	90%	3%
GLI3	cg07104748	Island	TSS1500	0.0463	0%	2%	2%	0%	1%	1%
GLI3	cg12853563	Island	TSS1500	0.0256	5%	9%	4%	8%	6%	2%
HSD11B1	cg06828613		5′UTR	0.0283	45%	62%	17%	55%	48%	7%
MATN3	cg10334703	S_Shore	TSS1500	0.037	15%	37%	23%	30%	20%	10%
SMO	cg10586510	Island	Body	0.0423	9%	14%	5%	13%	10%	3%
SMO	cg06217803	S_Shore	Body	0.0111	74%	84%	10%	76%	79%	3%

## Discussion

Babies born at lower gestational ages and birth weights carry an increased susceptibility for a range of diseases [29], [30], [31], [32], [33], [34], although there is considerable variation in outcome at the level of the individual. Prognostic markers, such as expression levels of candidate genes, might predict which individuals are on a developmental path towards a higher risk of disease, particularly as these trajectories need not involve children of abnormal gestational age or birthweight. We were able to study gene expression pattern in umbilical cords over a broad birthweight range and explored the spectrum of near-term and term gestational ages, i.e. from 35 to 41 weeks by leveraging the availability of birth tissue specimens collected within the Singapore GUSTO cohort study.

Our study was designed to investigate the molecular correlates of both birthweight and gestational age. Perhaps surprisingly, considering the dominant focus on birth weight, we found that gestational age (even when excluding prematurity), is a very important factor driving the transcriptome. Birthweight, even at extremes, is not. This finding is supported by two recent papers sampling umbilical cord blood from Caucasian and African-American mothers in the Conditions affecting Neurocognitive Development and Learning in Early Childhood (CANDLE) cohort. Adkins et al (2012) [35] failed to find significant associations with birth weight at either the transcript or CpG methylation level. However, Schroeder et al (2011) [28] found significant associations of CpG methylation with gestational ages, within the normal range. Our conclusion also is supported by the work of Cohen et al (2007) [36] who also found transcriptome-wide statistical associations with gestational age. Finally, at the functional level, morphology in specific brain regions at 6-years of age is better predicted by gestational age than by birth weight [37]. Our findings are in contrast to those of Mason et al (2010) [38] who found the largest component of variation in the umbilical cord blood transcriptome of neonates was not related to gestational age. Differences in tissues and sample sets may explain this inconsistency.

Our study suggested differential expression of the Hedgehog pathway by gestational age. This conclusion is the result of statistical analysis of the microarray data and only genes GLI2, GLI3, SMO, COL1A1 and MATN3 have been independently validated in the expanded qPCR sample set. The Hedgehog pathway has been shown to affect chondrocyte and osteoblast differentiation [39], [40]. Fetal bone development accelerates in late pregnancy and has a complex relationship with birthweight and gestational age [41], [42]. Lower gestational age and lower birth weights are associated with lower bone mass in infancy and adulthood [1], [43] and possibly with later development of osteoporosis [44], [45]. Lewis et al (2012) [46] reported that a higher placental PHLDA2 expression was associated with a lower fetal femur growth velocity. Dennison et al (2001) [47], found that the relationship between lumbar spine bone mineral density and birthweight varied according to vitamin D receptor genotype.

In addition, we saw the differential expression in the microarray data only, of the hedgehog target genes *PITX1*, *MATN3*, and *RUNX2.* Expression of transcription factor *PITX1* has been shown during hind limb development in regions giving rise to cartilage joints, long bones and skeletal muscles, while its partial inactivation led to a progressive formation of osteoarthritis-like phenotype in aging *Pitx1*+/− mice [48]. Polymorphisms in *PITX1* have also been recently associated with osteoarthritis in a Chinese population [49]. *MATN3* is a cartilage-specific matrix protein, mutations in which result in early-onset osteoarthritis [50]. The *RUNX2* transcription factor is also downstream of the hedgehog pathway and necessary for chrondrocyte and osteoblast differentiation and bone formation [51]. During osteoblast differentiation, *RUNX2* upregulates the expression of bone matrix protein genes including *COL1A1, COl1A2* [52] and the BMP family [52], which were also differentially expressed for gestational age in our study. Mutations in COL1A1 and COL1A2 have implicated in the inheritance of osteogenesis imperfecta and have a relationship with bone mineral density in Chinese populations [53].

Other genes differentially expressed in umbilical cords in relationship to gestational age and replicated in our qPCR study fall into the cytokine and angiogenesis pathways. *CXCL14* belongs to the family of CXC cytokines with a function in monocyte activation [54]. It has recently been associated with regulating metabolism, as it was shown that overexpression enhances obesity induced insulin resistance [55], [56]. In contrast, *CXCL14*−/− knockout mice are leaner and have a lower body weight [57]. Increased expression levels of *CXCL14* in ≤37 week gestation babies may thus trigger a trajectory towards insulin resistance later in life [7], [58]. Interestingly, *CXCL14* expression has been associated with reactive oxygen species, whereby downregulation of *CXCL14* leads to a stimulation of angiogenesis in head and neck cancer [59]. The relatively high levels of *CXCL14* in umbilical cord from ≤37 week gestation babies may reflect a restrictive intrauterine environment such as a dysfunctioning placenta [60]. The protein *IL1RL1* belongs to the interleukin 1 receptor family [61]. It is involved in eosinophilic inflammation which is linked to the allergic phenotype [62]. Moreover, polymorphisms in the *IL1RL1* gene are associated with the onset of asthma in Caucasian populations [63]. In our studies, we found a decreased mRNA expression of *IL1RL1* in cord tissue derived from Asian babies born at lower gestational age. We do not know the functional impact of this down-regulation of mRNA expression of *IL1RL1* and whether there is an overshooting compensatory expression later in development. *IL-33* is the natural ligand for *IL1RL1* and promotes angiogenesis by stimulating endothelial NO synthase [64]. Therefore, a decrease in NO production is directly linked to lowered expression of the receptor for *IL-33* (*IL1RL1*) and could reflect a supporting causative factor in the etiology of fetal growth restriction. Endothelial NO synthase (*eNOS*) is the primary isoenzyme expressed in human placenta but was found to be expressed in umbilical cord as well [65]. Insufficient nitric oxide production may associate with the pathogenesis of preeclampsia, a condition often leading to preterm birth. The activity of NO synthase in women with placental insufficiency was reported to be below normal levels [66].

Fetal development requires efficient umbilical blood flow, which in turn depends on the fetal vascular tree within the placenta. Enhanced angiogenesis influences neonatal birth weights and pregnancy outcomes (reviewed in [67]). We saw decreased expression of *ANTXR2* in umbilical cords of less than 37 weeks gestation babies, *ANTXR2* was recently reported to promote endothelial proliferation and morphogenesis during sprouting angiogenesis [68].

The Hedgehog signalling pathway has many functions, but separate from its function in bone formation (see above), the Hedgehog signalling pathway also plays a role in angiogenesis [69]. Using a model of mouse hind limb ischemia, Benameur et al. have shown that the hedgehog pathway promotes neovascularisation via the activation of *eNOS* and therefore increased nitric oxide production [70]. It is tempting to speculate about the nature of hedgehog pathway activation at lower gestational ages and whether activation of this pathway can be seen as a compensatory mechanism to counteract the generally anti-angiogenic gene-expression footprint observed in the ≤37 weeks gestation neonates.

Another gene of interest expressed at decreased levels in the ≤37 weeks gestation group was *HSD11B1*. *HSD11B1* is a bidirectional enzyme most associated with converting the inactive metabolite cortisone to the cortisol, which promotes insulin resistance and obesity [71]. Moreover, polymorphisms in *HSD11B1* have been linked to insulin resistance and metabolic syndrome in multiple populations [72], [73]. Transcript levels have been shown to be elevated in fetal membranes at late gestation [26] and to increase in mouse liver in response to high fat diets, leading to greater levels of insulin and hepatic lipid accumulation [74]. Both known hydroxysteroid-dehydrogenase isoforms HSD11B1 and HSD11B2 play a crucial role in maintaining physiological levels of maternal stress-induced glucocorticoids, and dysregulation of enzyme activity can lead to IUGR in rodent models [75] as well as human pregnancies [76]. The overexpression of isoform *HSD11B1* in >37 weeks gestation neonates is in line with published observations [26]. It is tempting to speculate whether enzymatic activities of the HSD11 isoforms are changed in a gestational age dependent manner, as this has been demonstrated in children born small for gestational age with lack of catch-up growth who were found to have lower activity of *HSD11B1* [77].

We suggest that differential expression observed in the umbilical cord transcriptome corresponding to gestational age is partly driven by specific DNA methylation changes. Schroeder et al (2011) [28] found that gestational age within the normal range significantly drives DNA methylation. Interestingly one of the CpGs which reached experiment-wide significance in their study was located in *GLI3*, a gene with transcript levels significantly varying with gestational age in our study. We have also measured the methylation state of the umbilical cords in our study and report concordant relationships with DNA methylation for 8 of the 16 genes replicated in our qPCR study as having significant concordance with gestational age at the expression level, including hedgehog transcription factor *GLI3*.

DNA methylation is an epigenetic mechanism that could explain the persistence of birth outcomes on disease susceptibility in later life [78]. Relton et al (2012) [79] recently reported DNA methylation variance in nine genes at birth, persisting to gene expression differences at 11–13 years of age and associating with body composition at nine years of age. Specific methylation differences correlating to gestational age could drive both the transcriptome of the umbilical cord at birth and have consequences in specific tissues at later ages. We chose here to assay the genomics of umbilical cord tissue as the only available somatic tissue. It is unknown if the genomic state of the umbilical cord correlates with that of the other tissues of the fetus, especially in those that give rise to diseases that have developmental origins. However, the available data suggests that DNA methylation patterns are generally conserved across tissues and there are examples of individually-variable epigenetic marks being soma-wide [80]. Godfrey et al (2011) [9] have shown that methylation of a site in the RXRα gene promoter in the umbilical cord correlates with body composition in later life, suggesting widespread influences across multiple tissues. It is a limitation of our study that results on whole umbilical cord tissue cannot be attributed to specific cell lineages. However, by using this heterogeneous tissue we anticipate that we have enriched for soma-wide changes.

In this study, we have made use of frozen umbilical cord tissue derived from an Asian population. By designing the study to independently examine both birth weight and gestational age, we were able to find expression changes significant for gestational age. Furthermore, by combining gene expression microarray data with corresponding data from the latest Infinium 450 K human methylation bead array chip (Illumina), our results provide insights into the epigenetic profile of babies with differing birth outcomes. This may allow the development of novel predictive epigenetic markers for non-communicable diseases prevalent in Asia.

## Materials and Methods

### Clinical Populations and Sample Collection

All specimens were from babies born at the KK Women’s and Children’s Hospital (KKH) and the National University Hospital (NUH), in Singapore. These hospitals are part of the GUSTO birth cohort study [25]. Written parental consent to participate in the study was given and hard copies are stored by the GUSTO data team. Ethical approval for the study and the consent forms and contents was granted, by the ethics boards of both KKH and NUH, which are centralised Institute Review Board (CIRB) and Domain Specific Review Board (DSRB), respectively. Gestational age was defined from a dating ultrasound (10–12 weeks) followed by an additional scan at 18–22 weeks.

The discovery microarray analysis sample set consisted of a total of 32 umbilical cords from babies of Chinese ethnicity. Maternal ages were restricted to between 20–40 years. It was designed to include eight low birth weight samples (defined as <2500 g), eight high birth weight (>3700 g) samples and sixteen normal birth weight babies with gestational ages matching the extreme birthweight samples. The average birth weight in the GUSTO cohort was 3081 g, which is comparable to the average across a larger Singaporean sample of 3183 g. for a term infant (unpublished data). All children had a gestational age in the range of 35–37 (shorter gestational age) or 38–41 weeks (longer gestational age), to allow comparison across the range of normal gestational age. This resulted in four groups, each comparable for birthweight and/or gestational age and matched for gender and maternal age.

The expanded replication sample set of 127 umbilical cords for qPCR analysis did not include any of those from the discovery set, and were from babies of Chinese, Indian and Malay ethnicities. The 20 samples analysed for DNA methylation were a subset of the discovery microarray analysis set of 32. [See Table 1 for sample characteristics]. Umbilical cord tissue samples were collected at the time of delivery, flushed with saline to remove fetal blood and flash-frozen in liquid nitrogen within 30 min of collection.

### RNA Extraction

Umbilical cord tissue (300 mg) was first placed in a sterile Dispomix tube and homogenized for 55 s for 3 cycles in 3 ml of Trizol using the Dispomix (Medic Tools, AG, Zug, Switzerland). After spinning down the debris, the supernatants were divided equally into three 2 ml tubes. 200 ul of chloroform were added to each tube, vortexed vigorously and centrifuged for 15 min at 4°C. The aqueous phase was carefully transferred to a new tube containing 1 ul of linear acrylamide. An equal amount of isopropanol was added and mixed by inversion. After incubating at −20°C overnight to precipitate the RNA, the pellet was obtained by centrifuging at 13,200 rpm for 10 min at 4°C. The RNA pellet was washed twice in 70% (v/v) ethanol, air-dried and resuspended in RNase-free water. The isolated RNA was then purified using the RNeasy Mini Kit (Qiagen, Hilden, Germany). On-column DNase digestion was carried out before the first wash step according to the manufacturer’s instructions. The purified RNA was then eluted in 30 µl of RNase-free water and stored at −80°C. RNA concentration and purity were measured using a nanodrop ND-8000 spectrophotometer (Nanodrop Technologies, Wilmington, DE, USA), and RNA integrity was determined using the Agilent 2100 Bioanalyzer and RNA 6000 Nano Labchips (Agilent Technologies, Santa Clara, CA, USA).

### Expression Microarray

Gene expression analysis was carried out with 3 sets of duplicate technical replicates from the 32 study subjects. All subsequent experimental steps followed the manufactures instructions. Briefly, Cy3-labelled cRNA was generated from 100 ng of total RNA using the Quick Amp Labelling Kit (One-Color) (Agilent Technologies, Santa Clara, CA, USA). Hybridization performance was assessed by means of 10 proprietary spike-in controls incorporated into the cRNA synthesis procedure. The labelled cRNA was then purified and hybridized onto Agilent SurePrint G3 Human Gene Expression (8×60 K) microarrays in a rotating (10 rpm) hybridization oven for 17 h at 65°C, after which they were washed and processed with proprietary buffers and solutions. The microarrays were then scanned at a resolution of 3 µm on an Agilent scanner using an extended dynamic range (PMT 10/100). The image data were processed using default values in feature extraction version 10.7.1.1 (Agilent Technologies, Santa Clara, CA, USA).

Agilent “.txt” files were outputted from the scanner and loaded into Arraystudio (Omicsoft). Signal extraction was performed from the gProcessedSignal value incorporating background subtraction. All expression values were log transformed. All probes with expression levels less than two standard deviations above background were removed. Values across replicate probes were averaged. Data were normalised amongst samples using quantile normalisation. Two samples with MAD scores <−5 were removed from the analysis. Duplicate data for the same sample were averaged. Data were subjected to principal component analysis and unsupervised hierarchical clustering (correlation distance, complete link). To identify probes significantly correlated with gestational age, both univariate regression and one-way ANOVAs (multiple testing correction: Benjamimi Hochberg) were performed.

Processed and raw data is deposited in GEO, series accession = GSE37100.

### Quantitative Real Time-PCR

erse transcribed using a High Capacity cDNA Reverse Transcription Kit (Applied Biosystems Inc, ABI, CA, USA). PCR reactions were prepared using 10 ul of Power SyBr Green PCR 2× Master mix (Applied Biosystems Inc, ABI, Foster City, CA, USA), 1 ul of each primer (2 uM), and 20 ng of cDNA in a total reaction volume of 20 ul. One hundered and twenty seven new samples were selected and PCR for each sample was done in triplicates in 384-well plates using the ABI 7900 HT Sequence Detection System. Cycle parameters used were 10 min at 95°C (1 cycle), then 15 s at 95°C and 1 min at 60°C (40 cycles). A dissociation step was added at the end of each run to check for amplification specificity. Twenty-two target genes and two endogenous control genes (GAPDH and beta-actin) were analyzed. The characteristics of the samples are shown in **Table1**. The 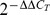 [81] was applied to calculate relative quantification of target genes. For each target gene, the threshold cycles (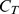) of samples provided from the equipment software (SDS 2.4) were normalized by the average 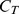 of both controls using 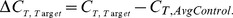. A sample (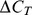 is very close to the group mean) from >37 weeks gestation group was then selected as the reference and then the 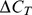 of all samples were normalized by this reference using 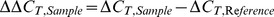. Finally, the fold changes of all samples were calculated by 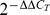. All samples were grouped as either <37 weeks gestation or as >37 weeks gestation. The differences between the two groups were examined by one way ANOVA.

### Pathway and Network Analysis

Pathway enrichment and *de novo* network discovery were performed in GeneGO metacore [82]. Pathway enrichment was calculated using a hypergeometric distribution against both Gene Ontologies and GeneGo pathway maps. Results were corrected for FDR using Benjamini-Hochberg. Network discovery was performed using the “shortest path” and “direct interactions” module. Low confidence, indirect and “influence by expression” interactions were pre-filtered and canonical pathway interactions were retained.

### Illumina® Infinium® HD Genome-wide Methylation Assay

Genomic DNA methylation analysis was carried out with 3 sets of duplicate technical replicates from 20 study subjects. All subsequent experimental steps followed the manufacturers’ instructions. After extraction of genomic DNA from frozen umbilical cord specimens according to standard procedures, 1 mg was bisulfite converted using EZ-96 DNA Methylation™ Gold Kit (Zymo Research, Irvine, CA, USA). Successful conversion was confirmed via methylation-specific PCR prior to proceeding with subsequent steps of the Infinium assay protocol. The bisulfite converted genomic DNA was isothermally amplified at 37°C for 22 hrs, enzymatically fragmented, purified and hybridized on an Infinium® HumanMethlyation 450 BeadChip (Illumina Inc., San Diego, CA, USA) at 48°C for 18 hrs. After which, the BeadChip was then washed to remove any un-hybridized or non-specific hybridized DNA. Labelled single-base extension was performed on primers hybridized with DNA, and the hybridized DNA was removed. The extended primers were stained with multiple layers of fluorescence, the BeadChip was then coated using a proprietary solution and scanned using the Illumina® iScan system. The image data were processed with the Genome Studio™ Methylation Module software.

The intensity files (.idat) produced by the Illumina iSCAN system were loaded into GenomeStudio’s methylation module for signal extraction. Background subtraction was performed by averaging the signals from the internal negative control beads. CpGs with less than three beads for either probe for any sample (18,603), or with signal detection *p-*values (calculated from the individual bead intensities) less than 0.05 (2,949) for any sample, were discarded for all samples. This step removed 4.4% of the 485,577 CpGs assayed. Data were normalized to the internal controls, which were designed to be housekeeping genes with no CpGs in the probe (samples the variation inherent in the array). β-values were then calculated, which are the ratio of the methylated probe intensity and the overall intensity. The β-value for an i^th^ interrogated CpG site was calculated by:
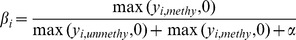



Where 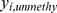 and 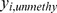 are the intensities measured by the i^th^ methylated and unmethylated probes respectively, averaged over the replicate beads, and “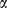 ” is a constant offset(by default 100). Therefore β -values range between 0–1, with 0 representing no methylation and 1 representing 100% methylation.

Tables of CpG β-values across samples were exported from GenomeStudio and loaded into Arraystudio for downstream analysis. As a further QC step MAD scores were calculated for the sample sets. MAD is a robust measure of statistical dispersion and is defined as the median of the absolute deviations from the data’s median:
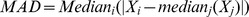


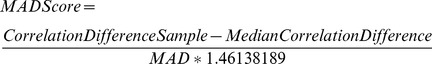



Samples with a MAD score of less than −5 were discarded. Principal component analysis and hierarchical clustering were performed to observe the clustering of technical replicates and discernible batch effects. No batch effects were observed. The intra-sample deviation was lower than the inter-sample deviation. Regression analysis was performed against gestational age to identify CpGs whose methylation levels co-varied. CpGs with a regression p<0.05, mapping to a gene whose differential expression by gestational age was replicated, were reported. No CpGs passed a Benjamin-Hochberg correction for multiple testing.

## Supporting Information

Figure S1 **Hierarchical clustering of the full data set shows that replicates cluster together.** However, there is no discernable clustering by birthweight or gestational age group. Each sample is represented as a column in the heatmap and probe by a row in the heatmap. Heatmap colouring is on normalised expression level (green = low, white = intermediate, red = high). Dendogram of sample clustering is above the heatmap. Colour bars showing sample group (HBW =  gold, LBW = green, ≤37w_NBW = blue, >37w_NBW = red) and gestational age (green  =  low, white = intermediate, red  = high).
(TIF)

Figure S2 **Tests for differential expression by gestational age return transcript levels co-varying with gestational age and significantly different between gestational age groups. A and B,** Examples of significantly co-varying transcript level by gestational age: probe A_23_P209246, mapping to the *GLI2* (A) and probe A_23_P70818, mapping to the *SMO* (B) log2 expression levels across samples are represented on the y-axis and gestational ages of those samples are represented on the x-axis. Samples with gestational age ≤37 weeks are denoted in blue, >37 weeks in red. **C and D**, Results from 1-way ANOVA tests for transcripts whose expression levels are significantly different between samples with gestational age ≤37 weeks and samples with gestational age >37 weeks. Average differences in expression levels between the two gestational age groups are represented on the x-axis, −log10 pvalues from the ANOVA tests are represented on the y-axis. Probes above the horizontal red line have nominal pvalues <0.05. Probes in the black box in C have FDR corrected pvalue<0.05. Transcripts mapping the genes mentioned in the text are highlighted in red and labelled. C, contains only samples of normal birthweight i.e. ≤37w_NBW vs. >37w_NBW. D, includes all samples i.e. LBW and ≤37w_NBW vs. >37w_NBW and HBW.
(TIF)

Figure S3 **Example of significantly co-varying transcript level by birthweight: probe A_33_P331451 (mapping to the** ***TGFBR1*** **) log2 expression levels across samples are represented on the y-axis and birth weights of those samples are represented on the x-axis.** LBW samples are denoted in green, NBW samples in purple and HBW samples in blue.
(TIF)

Table S1 **Probes whose expression levels co-varied with gestational age with a p<0.001 by Pearson’s regression.**
(XLSX)

Table S2 **Probes whose expression levels co-varied with birth weight with a p<0.001 by Pearson’s regression.**
(DOCX)

Table S3 **Probes whose expression levels were significantly different between gestational age groups with FDR corrected pvalue of <0.005.**
(DOCX)

Table S4 **Probes whose expression levels co-varied with birth weight (p<0.001 by Pearson’s) and were significantly different between birth weight groups (p<0.05 by ANOVA) and had a fold change between birthweight groups of >1.5.**
(DOCX)

Table S5 **20 genes studied in expanded sample set of 120 by qPCR.** The replicating group comprises genes whose mRNA levels had a significant relationship with gestational age in the qPCR expanded study. The non-replicating group are those genes whose mRNA levels did not achieve significance against gestational age in the expanded qPCR study.
(DOCX)

Table S6 **Genes containing probes whose transcript levels were significantly different between birthweight groups in the microarray analysis and also containing CpGs whose methylation levels correlated with birthweight in the Infinium analysis.**
(DOCX)

Text S1 **Detailed description analysis of correlation between mRNA level and DNA methylation state across samples paired fro gestational age. ** Four CpGs from table 2 in CHRDL2, GLI2 and HSD11B1 had significant (p<0.05) correlation of GA ratios between RNA expression and DNA methylation, when all the samples were used to create 17 non-unique pairs.
(DOCX)
