# Supplementary figures and images for: Transcriptome Changes Affecting Hedgehog and Cytokine Signalling in the Umbilical Cord: Implications for Disease Risk
Source: PLoS One. 2012 Jul 10;7(7):e39744. doi: 10.1371/journal.pone.0039744 (PMC3393728; doi:10.1371/journal.pone.0039744)

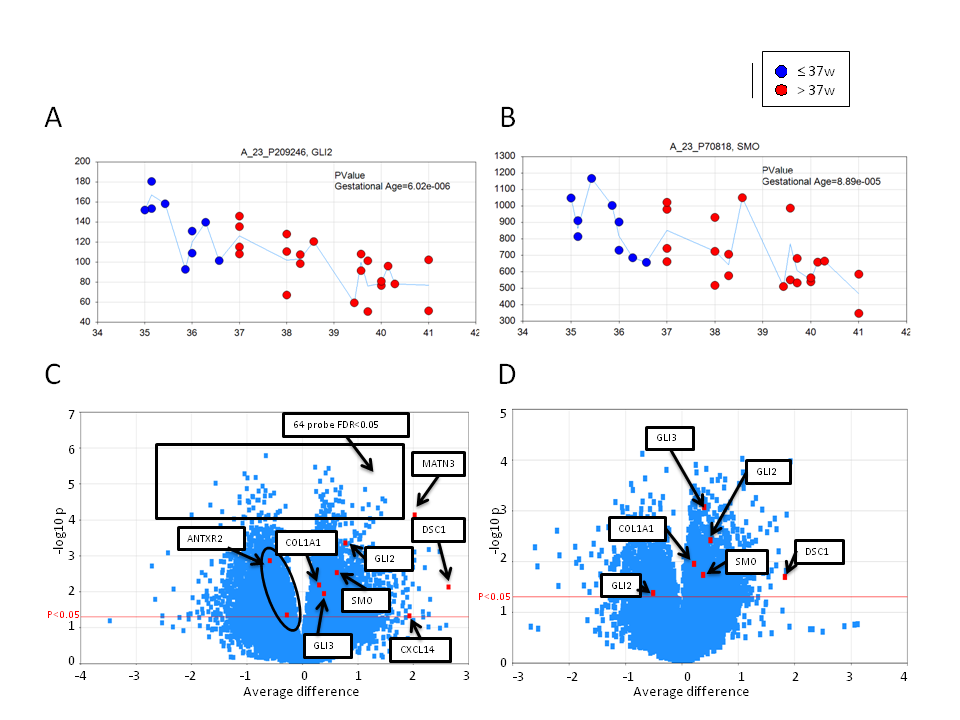

Supplement: Figure S2 — Tests for differential expression by gestational age return transcript levels co-varying with gestational age and significantly different between gestational age groups. A and B, Examples of significantly co-varying transcript level by gestational age: probe A_23_P209246, mapping to the GLI2 (A) and probe A_23_P70818, mapping to the SMO (B) log2 expression levels across samples are represented on the y-axis and gestational ages of those samples are represented on the x-axis. Samples with gestational age ≤37 weeks are denoted in blue, >37 weeks in red. C and D, Results from 1-way ANOVA tests for transcripts whose expression levels are significantly different between samples with gestational age ≤37 weeks and samples with gestational age >37 weeks. Average differences in expression levels between the two gestational age groups are represented on the x-axis, −log10 pvalues from the ANOVA tests are represented on the y-axis. Probes above the horizontal red line have nominal pvalues <0.05. Probes in the black box in C have FDR corrected pvalue<0.05. Transcripts mapping the genes mentioned in the text are highlighted in red and labelled. C, contains only samples of normal birthweight i.e. ≤37w_NBW vs. >37w_NBW. D, includes all samples i.e. LBW and ≤37w_NBW vs. >37w_NBW and HBW. (TIF) [file pone.0039744.s002.tif]
