# Supplementary material for: Transcriptome Changes Affecting Hedgehog and Cytokine Signalling in the Umbilical Cord: Implications for Disease Risk
Source: PLoS One. 2012 Jul 10;7(7):e39744. doi: 10.1371/journal.pone.0039744 (PMC3393728; doi:10.1371/journal.pone.0039744)
Supplement: Table S2 — Probes whose expression levels co-varied with birth weight with a p<0.001 by Pearson’s regression. (DOCX) [file pone.0039744.s005.docx]

**Supplementary table S2 – Probes correlated with BW p<0.001**

| probeid | Covariate | Correlation | Correlation PValue | Gene Symbol | GeneName |
| --- | --- | --- | --- | --- | --- |
| A_19_P00317789 | BW | -0.6212 | 0.0002 |  |  |
| A_23_P21134 | BW | 0.6164 | 0.0003 | DDIT3 | DNA-damage-inducible transcript 3 |
| A_23_P303145 | BW | -0.5868 | 0.0007 |  |  |
| A_23_P397308 | BW | -0.653 | 9.15E-05 | KLC4 | kinesin light chain 4 |
| A_23_P419150 | BW | 0.5742 | 0.0009 | VENTX | VENT homeobox homolog (Xenopus laevis) |
| A_33_P3331451 | BW | 0.5784 | 0.0008 | TGFBR1 | "transforming growth factor, beta receptor 1" |
| A_33_P3383687 | BW | -0.5858 | 0.0007 | LOC100132588 | hypothetical protein LOC100132588 |
