# Supplementary material for: Transcriptome Changes Affecting Hedgehog and Cytokine Signalling in the Umbilical Cord: Implications for Disease Risk
Source: PLoS One. 2012 Jul 10;7(7):e39744. doi: 10.1371/journal.pone.0039744 (PMC3393728; doi:10.1371/journal.pone.0039744)
Supplement: Table S3 — Probes whose expression levels were significantly different between gestational age groups with FDR corrected pvalue of <0.005. (DOCX) [file pone.0039744.s006.docx]

**Supplementary table 3 - 64 probes which passed an FDR correction for multiplicity of q<0.05, when gestational age categories were compared**

| **probeid** | **Average <37w_NBW**  **(log2)** | **Average >37w_NBW**  **(log2)** | **<37w_NBW vs. >37w_NBW pvalue-FDR_BH** | **GeneSymbol** |
| --- | --- | --- | --- | --- |
| A_33_P3310929 | 10.6274 | 9.5013 | 0.0315 | ADAM12 |
| A_23_P146728 | 8.7629 | 8.1757 | 0.0487 | ALG2 |
| A_33_P3380837 | 13.5396 | 14.2572 | 0.0315 | AMZ1 |
| A_33_P3280040 | 4.9992 | 5.9618 | 0.0315 | ANKRD11 |
| A_23_P122906 | 10.0245 | 9.3163 | 0.0414 | AUTS2 |
| A_23_P148015 | 10.2573 | 9.5983 | 0.0315 | AXIN2 |
| A_33_P3365878 | 13.3005 | 13.9527 | 0.0409 | BMP8B |
| A_23_P14649 | 12.3998 | 13.3024 | 0.0315 | C15orf28 |
| A_33_P3326578 | 1.7109 | 3.2762 | 0.0315 | C2orf55 |
| A_23_P18055 | 8.1526 | 9.0432 | 0.0315 | C3orf51 |
| A_32_P4985 | 5.8034 | 4.4459 | 0.0366 | CAMTA1 |
| A_24_P278747 | 13.1091 | 12.4155 | 0.0315 | CCND2 |
| A_23_P368886 | 7.9451 | 6.7532 | 0.0337 | CHSY3 |
| A_23_P384044 | 3.9811 | 2.4889 | 0.0315 | CNIH3 |
| A_33_P3422728 | 6.9844 | 8.1172 | 0.0315 | CNTNAP3 |
| A_23_P16722 | 8.9645 | 8.2326 | 0.0315 | DOCK10 |
| A_24_P299318 | 10.6635 | 9.77 | 0.0487 | FAM101B |
| A_33_P3390284 | 4.8215 | 5.5875 | 0.0464 | FAM122B |
| A_23_P214026 | 11.1943 | 9.7543 | 0.0315 | FBN2 |
| A_23_P213247 | 11.9523 | 11.3593 | 0.0315 | FBXL5 |
| A_33_P3380797 | 5.3748 | 6.2169 | 0.0315 | FGF3 |
| A_33_P3324333 | 8.6099 | 9.6889 | 0.0487 | FLJ43315 |
| A_23_P256641 | 6.3407 | 4.9228 | 0.0401 | KCNE1L |
| A_33_P3240507 | 12.5243 | 11.911 | 0.0409 | KCTD12 |
| A_33_P3236441 | 11.2702 | 12.403 | 0.0315 | LDHAL6A |
| A_33_P3212823 | 14.9119 | 15.6802 | 0.0401 | LOC100132247 |
| A_23_P117971 | 13.819 | 14.4889 | 0.043 | LOC100287593 |
| A_33_P3760125 | 12.4026 | 13.1954 | 0.0401 | LOC159110 |
| A_24_P229726 | 7.3379 | 6.4414 | 0.0337 | LOC440181 |
| A_23_P102058 | 4.7125 | 2.6892 | 0.0401 | MATN3 |
| A_23_P164057 | 15.2248 | 14.579 | 0.0401 | MFAP4 |
| A_33_P3351934 | 11.9682 | 12.8829 | 0.0464 | MSTO2P |
| A_23_P110430 | 9.1937 | 8.1049 | 0.038 | MSX1 |
| A_33_P3389917 | 5.5097 | 6.9561 | 0.0396 | MTR |
| A_24_P391586 | 6.98 | 5.7626 | 0.043 | OAF |
| A_33_P3240328 | 10.2845 | 9.6579 | 0.043 | PITX1 |
| A_33_P3310293 | 10.6186 | 11.2937 | 0.0315 | PKIG |
| A_23_P206059 | 8.8928 | 8.1666 | 0.0401 | PRC1 |
| A_23_P22433 | 10.0072 | 9.4057 | 0.0315 | RP2 |
| A_23_P419795 | 8.0958 | 7.3315 | 0.0401 | SBF2 |
| A_33_P3326349 | 5.9243 | 6.738 | 0.0315 | SMG5 |
| A_23_P156890 | 6.5244 | 5.2491 | 0.0467 | TCF21 |
| A_23_P150325 | 7.7329 | 6.9562 | 0.0487 | TMEM133 |
| A_23_P31143 | 12.4501 | 11.78 | 0.0315 | TPD52L1 |
| A_24_P201879 | 7.7534 | 7.079 | 0.0315 | UTP14C |
| A_19_P00316286 | 6.4612 | 7.3462 | 0.0366 |  |
| A_19_P00316801 | 7.9448 | 9.0075 | 0.0412 |  |
| A_19_P00321202 | 7.3416 | 8.388 | 0.0315 |  |
| A_19_P00321570 | 6.2148 | 6.8275 | 0.0401 |  |
| A_19_P00322966 | 6.8022 | 7.4675 | 0.0315 |  |
| A_19_P00323011 | 5.1564 | 6.0652 | 0.0468 |  |
| A_19_P00805180 | 5.3779 | 6.3839 | 0.0315 |  |
| A_19_P00807103 | 3.6445 | 4.8818 | 0.043 |  |
| A_19_P00808749 | 2.8745 | 4.8705 | 0.0401 |  |
| A_19_P00810982 | 6.2035 | 6.9378 | 0.0401 |  |
| A_19_P00813109 | 5.6991 | 6.4887 | 0.0315 |  |
| A_33_P3228722 | 7.0672 | 7.8563 | 0.0315 |  |
| A_33_P3235009 | 1.8303 | 3.4885 | 0.0337 |  |
| A_33_P3249716 | 7.3837 | 8.371 | 0.0315 |  |
| A_33_P3254306 | 4.4069 | 5.4395 | 0.0419 |  |
| A_33_P3268868 | 6.4263 | 7.4204 | 0.0315 |  |
| A_33_P3336449 | 1.7575 | 2.4466 | 0.0315 |  |
| A_33_P3352004 | 5.6841 | 6.3423 | 0.0315 |  |
