# Supplementary material for: Transcriptome Changes Affecting Hedgehog and Cytokine Signalling in the Umbilical Cord: Implications for Disease Risk
Source: PLoS One. 2012 Jul 10;7(7):e39744. doi: 10.1371/journal.pone.0039744 (PMC3393728; doi:10.1371/journal.pone.0039744)
Supplement: Table S4 — Probes whose expression levels co-varied with birth weight (p<0.001 by Pearson’s) and were significantly different between birth weight groups (p<0.05 by ANOVA) and had a fold change between birthweight groups of >1.5. (DOCX) [file pone.0039744.s007.docx]

**Supplementary table S4 – Probes correlated with BW p<0.05 and with p<0.05 and FC>1.5 in ANOVA tests**

| probeid | LBW vs. <37w_NBWFC | LBW vs. <37w_NBW p | HBW vs. >37w_NBW FC | HBW vs. >37w_NBW p | Correlation | Correlation  pValue | GeneSymbol | GeneName |
| --- | --- | --- | --- | --- | --- | --- | --- | --- |
| A_19_P00807356 | 1.86 | 0.0007 | -1.72 | 0.0024 | -0.4109 | 0.0241 |  |  |
| A_19_P00809574 | 1.67 | 0.0421 | -1.93 | 0.011 | -0.4593 | 0.0107 |  |  |
| A_23_P32577 | -1.88 | 0.0294 | 2.13 | 0.0102 | 0.4879 | 0.0062 | DACH1 | dachshund homolog 1 (Drosophila) |
| A_24_P63347 | -2.89 | 0.0337 | 3.35 | 0.017 | 0.4617 | 0.0102 | PF4V1 | platelet factor 4 variant 1 |
| A_33_P3269019 | 1.84 | 0.0031 | -1.89 | 0.0022 | -0.3661 | 0.0466 | LOC100128559 | hypothetical LOC100128559 |
| A_33_P3279353 | -3.81 | 0.0194 | 3.05 | 0.048 | 0.4157 | 0.0223 | AZU1 | azurocidin 1 |
| A_33_P3320533 | 1.64 | 0.0115 | -1.59 | 0.0179 | -0.4709 | 0.0086 |  |  |
| A_33_P3406661 | 1.66 | 0.0135 | 1.52 | 0.0373 | -0.3766 | 0.0402 | TMEM63C | transmembrane protein 63C |
