# Supplementary material for: Transcriptome Changes Affecting Hedgehog and Cytokine Signalling in the Umbilical Cord: Implications for Disease Risk
Source: PLoS One. 2012 Jul 10;7(7):e39744. doi: 10.1371/journal.pone.0039744 (PMC3393728; doi:10.1371/journal.pone.0039744)
Supplement: Table S5 — 20 genes studied in expanded sample set of 120 by qPCR. The replicating group comprises genes whose mRNA levels had a significant relationship with gestational age in the qPCR expanded study. The non-replicating group are those genes whose mRNA levels did not achieve significance against gestational age in the expanded qPCR study. (DOCX) [file pone.0039744.s008.docx]

Supplementary table 5:

| Name | Group | Description |
| --- | --- | --- |
| MDK | Replicating group | midkine (neurite growth-promoting factor 2) |
| GLI2 | Replicating group | GLI family zinc finger 2 |
| GLI3 | Replicating group | GLI family zinc finger 3 |
| COL1A1 | Replicating group | collagen, type I, alpha 1 |
| MATN3 | Replicating group | matrilin 3 |
| CXCL14 | Replicating group | chemokine (C-X-C motif) ligand 14 |
| DSC1 | Replicating group | desmocollin 1 |
| CHRDL2 | Replicating group | chordin-like 2 |
| SMO | Replicating group | smoothened, frizzled family receptor |
| IL1RL1 | Replicating group | interleukin 1 receptor-like 1 |
| HSD11B1 | Replicating group | hydroxysteroid (11-beta) dehydrogenase 1 |
| ANTRX2 | Replicating group | anthrax toxin receptor 2 |
| FOXP2 | Non-replicating group | forkhead box P2 |
| UBIAD1 | Non-replicating group | UbiA prenyltransferase domain containing 1 |
| PITX1 | Non-replicating group | paired-like homeodomain 1 |
| MSX1 | Non-replicating group | msh homeobox 1 |
| HDAC1 | Non-replicating group | histone deacetylase 1 |
| TIMP3 | Non-replicating group | TIMP metallopeptidase inhibitor 3 |
| BMP8B | Non-replicating group | bone morphogenetic protein 8b |
| TGFBR1 | Non-replicating group | transforming growth factor, beta receptor 1 |
| COL1A2 | Non-replicating group | collagen, type I, alpha 2 |
| GRID2 | Non-replicating group | glutamate receptor, ionotropic, delta 2 |
