# Supplementary material for: Transcriptome Changes Affecting Hedgehog and Cytokine Signalling in the Umbilical Cord: Implications for Disease Risk
Source: PLoS One. 2012 Jul 10;7(7):e39744. doi: 10.1371/journal.pone.0039744 (PMC3393728; doi:10.1371/journal.pone.0039744)
Supplement: Table S6 — Genes containing probes whose transcript levels were significantly different between birthweight groups in the microarray analysis and also containing CpGs whose methylation levels correlated with birthweight in the Infinium analysis. (DOCX) [file pone.0039744.s009.docx]

**Supplementary table 6:** Genes with differential transcript levels for birthweight in the microarray analysis, also containing CpGs whose methylation levels correlated with birthweight.

| **Gene Name** | **CpG ID** | **CpG location** | **UCSC_RefGene_Group** | **Correlation PValue** | **min beta value** | **max beta value** | **Min-max range** | **average beta for lowest GA samples** | **average beta for highest GA samples** | **Range between highest and lowest GA** |
| --- | --- | --- | --- | --- | --- | --- | --- | --- | --- | --- |
| DACH1 | cg13726218 | N_Shore | Body | 0.0355 | 18% | 31% | 13% | 29% | 24% | 5% |
| DACH1 | cg00071658 |  | Body | 0.0403 | 89% | 93% | 5% | 90% | 92% | 2% |
| DACH1 | cg23101138 | S_Shore | Body | 0.0455 | 80% | 89% | 9% | 87% | 87% | 1% |
| KLC4 | cg10177591 | Island | TSS1500 | 0.0219 | 8% | 11% | 3% | 10% | 9% | 1% |
| KLC4 | cg25550677 | Island | TSS1500 | 0.0431 | 2% | 17% | 15% | 5% | 12% | 6% |
| VENTX | cg22546168 | Island | TSS1500 | 0.0285 | 1% | 4% | 3% | 2% | 1% | 1% |
