# Supplementary material for: Transcriptome Changes Affecting Hedgehog and Cytokine Signalling in the Umbilical Cord: Implications for Disease Risk
Source: PLoS One. 2012 Jul 10;7(7):e39744. doi: 10.1371/journal.pone.0039744 (PMC3393728; doi:10.1371/journal.pone.0039744)

There was a significant correlation between mRNA levels as measured on the microarray and DNA methylation as measured on the Infinium array, across samples for a subset of CpGs in table 2. (qPCR samples were not analysed on the Infinium arrays)

For this analysis samples were paired, where one member of the pair had gestational age >37 weeks and one had gestational age ≤ 37 weeks. The pairs were matched for their gender and closest birthweight. The ratio of RNA expression values between the two GA groups were compared to the ratio of the DNA methylation values between the two groups, and their concordance were estimated by Spearman rank.

Four CpGs from table 2 in CHRDL2, GLI2 and HSD11B1 had significant (p<0.05) correlation of GA ratios between RNA expression and DNA methylation, when all the samples were used to create 17 non-unique pairs.


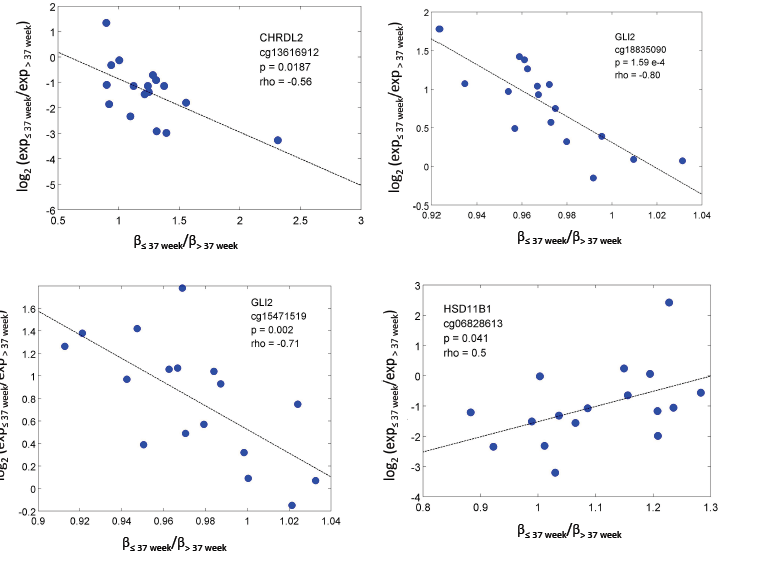


When the pairs were required to contain only unique samples – resulting in just 9 pairs in the 19 samples, three CpGs had significant correlations, all of them in GLI2:


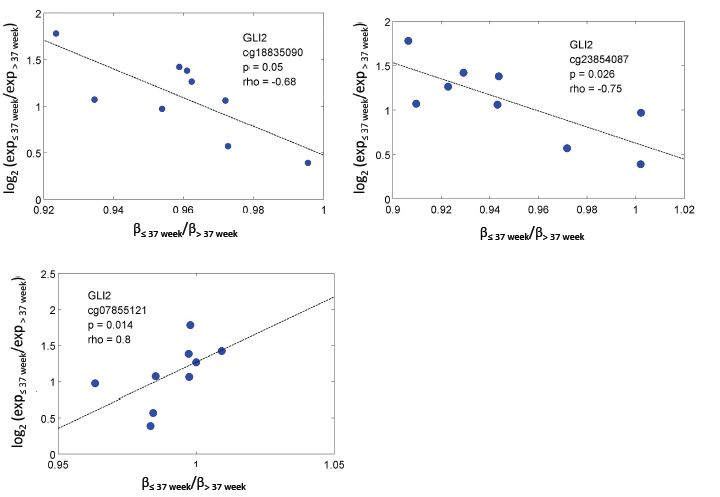


Finally, we investigated if there was a significant relationship across the samples for DNA methylation and RNA expression outside of the context of phenotype. Again we used spearman rank and five CpGs were significant, including three of the CpGs also significant in the previous with respect to GA analysis.


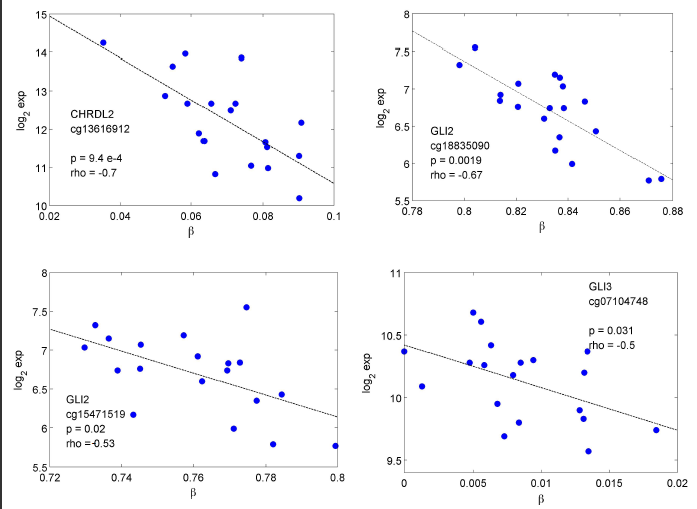


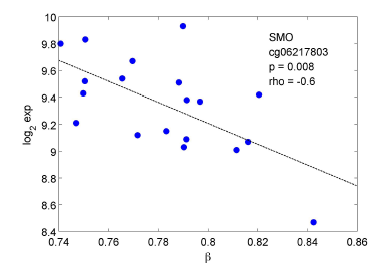

Supplement: Text S1 — Detailed description analysis of correlation between mRNA level and DNA methylation state across samples paired fro gestational age. Four CpGs from table 2 in CHRDL2, GLI2 and HSD11B1 had significant (p<0.05) correlation of GA ratios between RNA expression and DNA methylation, when all the samples were used to create 17 non-unique pairs. (DOCX) [file pone.0039744.s010.docx]
